# Polyarthrite chronique destructrice révélant une polychondrite atrophiante

**DOI:** 10.11604/pamj.2014.17.59.3574

**Published:** 2014-01-26

**Authors:** Faten Frikha, Zouhir Bahloul

**Affiliations:** 1 Service de Médecine Interne, CHU Hédi Chaker, 3029 Sfax, Tunisie

**Keywords:** Polyarthrite chronique, polychondrite atrophiante, chronic polyarthritis, atrophic polychondritis

## Image en medicine

La polychondrite atrophiante (PCA) est une affection rare caractérisée par une atteinte inflammatoire des cartilages avec une prédilection pour ceux du nez, du pavillon des oreilles et de l'arbre laryngotrachéobronchique. Des manifestations extracartilagineuses variées (rhumatologiques, oculaires, dermatologiques cardiovasculaires, audiovestibulaires ou rénales) sont possibles et parfois révélatrices. Patiente de 53 ans hospitalisée pour une polyarthrite déformante et destructrice et des épisodes d'ischémie digitale. A l'interrogatoire, on trouvait la notion de bourdonnement des oreilles avec diminution de l'acuité auditive. L'examen ostéo-articulaire révélait une atteinte déformante des mains et des pieds (A). L'examen clinique objectivait un effondrement de la pyramide nasale avec une destruction complète de la cloison cartilagineuse (B). La voix était dysphonique et il existait une surdité de perception bilatérale. A la biologie, il y avait un syndrome inflammatoire biologique avec une VS: 125 mm 1ère heure et une CRP à 49 mg/l. Le facteur rhumatoïde, les anticorps anti-CCP ainsi que les ANCA étaient négatifs. La radiographie des mains montrait une atteinte destructrice des MCP, des IPP et des IPD bilatérale et les images d'acro-ostéolyse de certaines phalanges (C). Le scanner du massif facial objectivait une Ostéolyse de la cloison nasale, un Comblement bilatéral du sinus maxillaire et des cellules ethmoïdales antérieures (D). Le diagnostic de PCA avec arthropathie destructrice était retenu. Un traitement par corticothérapie était instauré. L’évolution était partiellement favorable.

**Figure 1:**
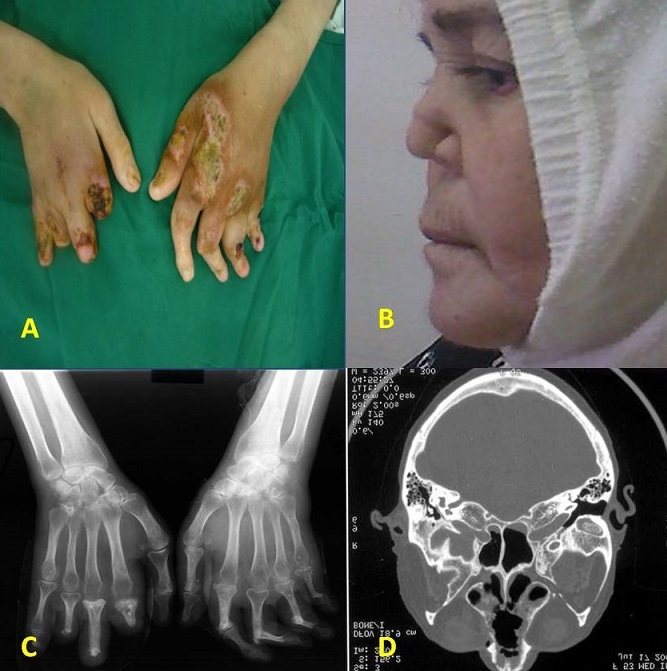
A)atteinte déformante des mains et présence de lésions ulcéro-nécrotiques de la main gauche; B)Effondrement de la pyramide nasale; C)radiographie des mains : une atteinte destructrice et des images d'acro-ostéolyse de certaines phalanges des doigts; D)Scanner du massif facial : Ostéolyse de la cloison nasale. Comblement bilatéral du sinus maxillaire et des cellules ethmoïdales antérieures

